# The Improved Quality of Gluten-Free Bread Due to the Use of Flaxseed Oil Cake: A Comprehensive Study Evaluating Nutritional Value, Technological Properties, and Sensory Quality

**DOI:** 10.3390/foods12234320

**Published:** 2023-11-29

**Authors:** Daniela Oliveira, Małgorzata Starowicz, Anita Ostaszyk, Łukasz Łopusiewicz, Isabel M. P. L. V. O. Ferreira, Edgar Pinto, Urszula Krupa-Kozak

**Affiliations:** 1 Institute of Animal Reproduction and Food Research, Polish Academy of Sciences, 10-748 Olsztyn, Poland; dsvmoliveira@gmail.com (D.O.); m.starowicz@pan.olsztyn.pl (M.S.); 2 LAQV/REQUIMTE, Laboratory of Bromatology and Hydrology, Department of Chemical Sciences, Faculty of Pharmacy, University of Porto, 4050-313 Porto, Portugal; isabel.ferreira@ff.up.pt (I.M.P.L.V.O.F.); ecp@ess.ipp.pt (E.P.); 3 Sensory Laboratory, Institute of Animal Reproduction and Food Research, Polish Academy of Sciences, 10-748 Olsztyn, Poland; 4 Center of Bioimmobilisation and Innovative Packaging Materials, Faculty of Food Sciences and Fisheries, West Pomeranian University of Technology, 71-270 Szczecin, Poland; lukasz.lopusiewicz@zut.edu.pl; 5 Department of Environmental Health, ESS, Polytechnic of Porto, 4200-072 Porto, Portugal

**Keywords:** oilseed cake, plant-based by-products, gluten elimination, bakery products, textural profile, sensory features, by-product revalorisation

## Abstract

The development of gluten-free bakery products, the quality of which is comparable to the quality of regular wheat-based products, remains a technological challenge. In this study, flaxseed oil cake (FOC), a by-product of flaxseed oil extraction and a source of nutritional and functional compounds, was used as an ingredient in the experimental bread formulation as partial replacement of starches (5%, 15%, and 30%). The gluten-free breads (GFBs) were evaluated for technological parameters, nutritional value, and sensory quality. Compared with the control, all FOC-enhanced GFBs were significantly (*p* < 0.05) richer in proteins, fat, and dietary fibre, with an increase that was proportional to the concentration of FOC in the formulation. At low-to-moderate levels (5% and 15%), FOC improved the specific volume, texture characteristics (reduced crumb hardness, gumminess, and chewiness), and appearance of GFBs, which allowed us to ameliorate its sensory features, but at 30% of FOC, the quality of the crumb texture decreased significantly (*p* < 0.005). Among the experimental GFBs, FOC15% exhibited improved technological characteristics and was rated by the sensory panel as the best in terms of overall quality. The results of the conducted research highlighted the benefits of incorporating FOC into GFB as a promising approach to developing a palatable, high-quality bakery product that may be a healthier option for individuals on a gluten-free diet, offering nutritional benefits. Nevertheless, it should be emphasised that the amount of FOC in GFB requires careful regulation.

## 1. Introduction

The production of vegetable oils generates significant amounts of residues, some of which are already recognised as by-products with specific applications, such as their use in animal feed production [1]. However, a portion of these residues continues to be discarded and contributes to environmental problems and economic losses in the plant oil industry. Exploring innovative uses and technologies for these underutilised residues can contribute to a more sustainable and circular economy [2]. Several studies have highlighted the potential benefits of a circular economy approach in the plant oil industry, including the reduction in waste and greenhouse gas emissions, as well as the creation of new business opportunities [3,4].

Flaxseed oil cake (FOC), a by-product of flaxseed oil extraction [5], has great potential to improve the nutritional and functional properties of food products. It offers a significant content of essential nutrients, including proteins (~32–36%) and fat (~12–21%), and is additionally abundant in dietary fibre (~9–10%) [6]. FOC proteins are characterised by a high content of essential amino acids, prominently arginine and leucine, and non-essential amino acids, with glutamic acid and aspartic acid being the major constituents [7]. Regarding the fat profile, FOC is particularly rich in polyunsaturated fatty acids, specifically linoleic acid (17%) and α-linolenic acid (52%) [7,8]. Moreover, FOC is also a good source of minerals (K, P, Mg, Zn), and bioactive compounds, such as lignans [6,7,9]. On the other hand, the potential benefits of FOC application in foods may be hindered by the presence of phytotoxic compounds, including phytic acids, cyanogenic glycosides, and linatine [10], which may reduce the bioavailability of nutrients or pose a health risk for consumers [11]. To make flaxseed derivatives safe for consumption, the antinutritive components must be removed or inactivated to undetectable limits. Phytic acid is a low molecular antioxidant [12]; however, it is considered antinutrient due to its ability to chelate with divalent cations (calcium, zinc, magnesium, copper, iron) and render them insoluble and unavailable for absorption [13]. Sourdough fermentation and leavening using yeast can help break down phytic acid in breadmaking due to the activation of native phytases, which release inorganic phosphate and a series of inositol phosphate intermediates. Baca et al. [14] indicated that the elevation of the temperature (up to 30 °C) and elongation of the time of yeast fermentation caused an increase in phytic acids hydrolysis due to an increase in phytase activity at the higher temperature. Contrary to phytic acid, cyanogenic glycosides are heat-labile and can be reduced or removed via thermal processing, solvent extraction, extrusion [15], and enzyme (β-glycosidases) application [16]. Thus, the combined techniques applied during breadmaking could be sufficient to reduce the content of antinutrients in FOC, enabling it to be used as a safe and valuable food ingredient. 

The use of FOC in different foods, along with its derivatives such as FOC extract [17] and FOC flour [18], has gained increasing attention due to its high nutritional value and potential applications as a functional ingredient in various food products. Many studies determined its effect on the physical, chemical, and sensory properties of the products, as well as the dietetic and potential health benefits associated with FOC consumption [17,18,19,20,21]. Łopusiewicz et al. [17] focused on the development and characterisation of a non-dairy kefir-like fermented beverage using FOC as subtract. The authors concluded that the FOC-based kefir-like beverage had a similar composition to traditional kefir and a higher content of probiotic bacteria, indicating its potential health-promoting effects. In addition, the authors showed a 94.05% reduction in cyanogenic compounds in FOC (from the primary amount of 187.35 ± 8.34 mg/kg to 11.15 ± 4.41 mg/kg) after incubating for 1 h at 90 °C, which is seen as a safe level for consumers [22]. Zarzycki et al. [19] developed an FOC-enriched pasta and assessed its nutritional value, antioxidant capacity, and cooking quality. The results showed a significant increase in all measured parameters, indicating the beneficial effects of incorporating FOC in pasta production. Regarding breadmaking, Taglieri et al. [20] conducted a study to examine the impact of using different leavening agents (sourdough and baker’s yeast) on the characteristics of bread that is fortified with FOC. Similarly, Sanmartin et al. [21] explored the use of FOC as an ingredient improving the nutraceutical and sensory features of sourdough bread.

FOC extract has also been applied to ameliorate the GFB quality. Krupa-Kozak et al. [23] investigated the impact of the level of FOC extract on the nutrient content, antioxidant properties, and sensory quality of GFB. The authors found that increasing the level of FOC extract in a formulation resulted in higher antioxidant activity, improved nutritional properties, and better sensory quality of the developed GFB. Meanwhile, Łopusiewicz et al. [24] evaluated the effect of FOC extract on the texture and shelf life of GFB, finding an improvement in these features. Moreover, FOC extended the shelf life of GFB and successfully delayed microbial growth, which could potentially increase the safety of GFB. All the above-mentioned studies have shown promising results relating to the applications of FOC in the food industry in terms of improving the nutritional value and sensory attributes of various food products. However, further research could be helpful to better understand the effects of FOC on GFB processing and quality characteristics.

Based on the literature, it is evident that commercial GFBs have nutritional limitations. In particular, these bakery products are recognised as being low in proteins and deficient in minerals (calcium, iron, and zinc) and vitamins (folate, niacin, thiamin, and riboflavin) that are needed in a healthy and balanced diet, in addition to being excessive in fat and simple sugars [25]. Therefore, it is important to enhance its nutritive value while simultaneously balancing this with technological and sensory benefits. On the other hand, the valorisation of plant-origin waste and by-products that are rich in nutrients, dietary fibre, and bioactive compounds into food recipes is a current trend in the development of value-added food products [23,24,26,27]. The main purpose of this study was to design and produce a new high-quality FOC-enriched GFB, characterised by improved nutritional features and enhanced sensory attributes. To examine the developed GFBs, the physical parameters, texture profile, proximal chemical composition, and sensory features were determined. 

## 2. Materials and Methods

### 2.1. Flaxseed Oil Cake

In the present study, FOC, produced and donated by ACS Sp. z o. o. (Bydgoszcz, Poland), was used. Our preliminary analysis (data not published) of FOC’s nutritional composition showed that it was a valuable source of proteins (30 g/100 g DM), carbohydrates (32 g/100 g DM), and fat (2.5 g/100 g DM). FOC was also rich in dietary fibre (7.9 g/100 g DM).

### 2.2. Composition of Experimental Gluten-Free Breads

The GFB used as the control was based on a previously optimised formulation [28] and was composed of corn starch (HORTIMEX, Konin, Poland), potato starch (PPZ “Trzemeszno” Sp. z o. o., Trzemeszno, Poland), pectin (E 440(i), ZPOW Pektowin, Jasło, Poland), sugar (Diamant, Pfeifer & Langen Polska S.A., Poznań, Poland), salt (Cenos Sp. z o. o., Września, Poland), fresh yeast (Lesaffre Polska S.A., Wołczyn, Poland), rapeseed oil “Wielkopolski” (EOL Polska Sp. z o. o., Szamotuły, Poland), and deionised water. FOC was added to the experimental formulation as a substitute for starches (Table 1). 

### 2.3. Preparation of Experimental Gluten-Free Breads

A straight dough method was used to prepare the experimental GFBs [28]. To make the GFBs, the main ingredients (starches, pectin, FOC) were mixed (5 min; t min. speed) using Kenwood Chef XL Titanium P-9878 (Kenwood Limited, Havant, UK). Subsequently, sugar, salt, and yeast dissolved in the deionised water were added to the mixture along with the oil. The batter was mixed at low speed (speed 2) for 12 min. Then, the batter was divided into 240 g samples and placed into the square pans (10 cm × 10 cm × 9 cm) and proofed for 40 min at 35 °C and 70% humidity. Afterwards, samples were baked in the oven (ZBPP, Bydgoszcz, Poland) for 30 min at 220 °C. Baked loaves were cooled for 2 h at room temperature and then stored (24 h) in the dark at room temperature in clip-seal plastic bags for further analysis. The products of four batches were analysed.

### 2.4. Sample Preparation for Further Analysis

To determine the moisture content, texture properties, and sensory analysis, fresh (24 h after baking) GFBs were used. On the other hand, the chemical composition and acrylamide content was determined in freeze-dried GFB samples. Briefly, a whole loaf of each type of GFB was manually crushed, packed in a paper envelope, and placed in the ultra-freezer at −80 °C for at least 24 h. Then, the frozen samples were placed in a freeze-dryer (Labconco Corporation, Kansas City, MO, USA) for about 40 h. The freeze-dried samples were ground with a laboratory mill (WZ-1 type, Zakład Badawczy Przemysłu Piekarskiego Sp. z o. o., Poland) for 12 s and sieved through a 0.40 mm mesh. The obtained homogenous powder was packed in polyurethane string bags and kept in the dark at 4 °C for further analysis.

### 2.5. Characteristics of Experimental Gluten-Free Breads

#### 2.5.1. Analysis of Nutritional Composition and Energy Value

Moisture [29], proteins [30], fat [31], ash [32], and dietary fibre [33] content were determined according to the standard methods. The content of carbohydrates was calculated by subtracting the values in percentage of moisture, fat, protein, and ash from 100. Energy values (kJ) were calculated as previously described [23]. The conversion factor for calorie calculation was considered to be 1 kJ = 0.239 kcal [34].

#### 2.5.2. Determination of Acrylamide Content

The acrylamide was extracted from gluten-free bread using the procedure of Ciesarová et al. [35] without modifications. Then, the micro-HPLC (LC-200, Eksigent) system coupled with a mass spectrometer (QTRAP 5500, AB Sciex, Vaughan, ON, Canada) consisting of a triple quadrupole and ion trap was used to analyse samples. The chromatographic separation was conducted on a HALO C_18_ column (0.5 mm × 50 mm × 2.7 μm, Eksigent, Vaughan, ON, Canada) at 45 °C at 25 μL/min flow rate. The elution solvents were A (H_2_O/formic acid; 99.9:0.1; *v/v*) and B (acetonitrile/formic acid; 99.9:0.1; *v/v*). The gradient elution was used as follows: 0–0.7 min (1% B), 0.7–3.2 min (1–90% B), 3.2–4.2 min (90% B), 4.2–4.4 min (90–1% B), and 4.4–5 min (1% B). A calibration curve with R^2^ = 0.998 was plotted for acrylamide using the external standard (17.4–1740 × 10^−1^ ng/g). The LOD and LOQ were established at the level of 2.54 × 10^−4^ µg/g and 0.77 × 10^−3^ µg/g, respectively. Acrylamide was identified and quantified by comparing its retention time and the presence of respective parent and daughter ion pairs (multiple reaction monitoring, MRM). Acrylamide (≥99%), acetonitrile, formic acid, water of MS grade, potassium hexacyanoferrate (II) trihydrate (K_4_[Fe(CN)_6_]**•**3H_2_O), zinc sulfate heptahydrate (ZnSO_4_**•**7H_2_O), and ethyl acetate were bought from Sigma Chemicals Co. (St. Louis, MO, USA).

#### 2.5.3. Determination of Physical Parameters

The loaf weight was determined using a digital balance (0.01 g accuracy), and its volume was determined using the standard rapeseed displacement method [36]. Three loaves of each GFB type were analysed.

Other physical parameters of experimental GFBs, in particular the specific volume (SV; cm^3^/g), density (D; g/mL), and the ratio of height to width (H/W), were determined as previously described [23], whereas the bake loss was calculated through Equation (1):

(1) $$Bake\;loss\;\left(\%\right)=\frac{\left(a-b\right)\times 100}{a}$$

where:

*a* is the weight of batter (g),

*b* is the weight of baked and cooled GFBs (g).

A middle slice of GFBs was scanned using a flatbed scanner (Epson Scan GT-1500, Epson Europe, Warsaw, Poland), supported by Epson Creativity Suite Software Images.

#### 2.5.4. Instrumental Colour Determination

Due to the irregularity of the crust surface of experimental GFBs, colour was analysed only in the crumb samples at the middle point of a central slice (of 20 mm thickness) using a Hunter Lab ColorFlex 45/0 (Hunter Associates Laboratory, Inc., Reston, VA, USA). The results were expressed following the CIELab system: lightness L* (=0 to black; =100 to white) and chromatic components a* (−*a* to greenness; +*a* to redness) and b* (−*b* to blueness; +*b* to yellowness). The whiteness index (WI) was calculated according to Hsu et al. [37]. The difference in colours (Δ*E_Lab_*), expressed as metric distances among the chromatic coordinates values [38], were calculated through Equation (2):

(2) $$\Delta E_{Lab}=\sqrt{{\left(\Delta L\right)}^{2}+{\left(\Delta a\right)}^{2}+{\left(\Delta b\right)}^{2}}$$

where

$$\Delta L=L_{1}-L_{0};\;\Delta a=a_{1}-a_{0};\;\Delta b=b_{1}-b_{0}$$

The crumb colour values for each kind of GFB were the mean of fifteen replications.

#### 2.5.5. Instrumental Textural Profile Analysis (TPA)

To analyse the texture of the crumb of GFBs, a TA.HD Plus Texture Analyser (Stable Micro Systems Ltd., Godalming, UK), equipped with a 30 kg load cell, was used. A 25 mm thick central slice was exposed to a double compression cycle up to 40% deformation of its original height with a 35 mm flat-end aluminium compression disc (probe P/35). The selected settings were as follows: pretest/test/post-test speed, 2.0 mm/s, force, 10 g, relaxation time, 5 s, trigger, and auto mode [39]. The textural parameters that were determined were as follows: hardness, springiness, cohesiveness, chewiness, and resilience. The texture profile was analysed in six replicates.

#### 2.5.6. Sensory Analysis

The trained and monitored according to the ISO standard [40] expert panel (five women and one man), acquainted with gluten-free products, performed the sensory analysis of experimental GFBs using quantitative descriptive analysis (QDA) [41]. The vocabulary for sensory attributes was determined in a round-table session, following the standardised procedure [42]. Twenty established attributes were defined, and the scale edges are shown in Table 2.

GFBs were evaluated using the QDA, which was performed in a sensory laboratory room [43] at room temperature and under normal lighting conditions. A three-digit number was assigned to each sample and given to the assessors all together in a random order. To minimise residual effects, water was available to drink between each sample evaluation. The panellists evaluated the intensity of attributes through unstructured graphical scales. Results were converted into numerical values (from 0 to 10 arbitrary units) via the ANALSENS system (IAR&FR PAS, Olsztyn, Poland). GFBs were tested in duplicate at different time points.

### 2.6. Statistical Analysis

In this study, unless specified otherwise, the results are shown as the mean of triplicate observations and standard deviation. The differences between experimental GFBs were analysed using one-way ANOVA, followed by Tukey’s multiple comparison test (*p* ≤ 0.05). The statistical analysis was conducted using GraphPad Prism version 8.0.0 for Windows (GraphPad Software; San Diego, CA, USA).

## 3. Results and Discussion

### 3.1. The Proximal Chemical Composition and Energy Value

The nutritional composition of the FOC and proximal chemical composition and energy value of the experimental GFBs are presented in Table 3. The obtained results showed that FOC, a by-product of flaxseed oil production, was a valuable source of proteins (30.46 g/100 g DM) and carbohydrates (32 g/100 g DM), and additionally, it was rich in fat (2.54 g/100 g DM) and dietary fibre (7.88 g/100 g DM). The comparable content of proteins (29.20 g/100 g) in flaxseed cake was recorded by Sanmartin et al. [21], whereas other studies confirmed that FOC was rich in dietary fibre [44], fat, and polyunsaturated fatty acids [8], especially α-linolenic acid, which has been shown to have beneficial effects on human health.

Previous studies have reported that conventional wheat bread gained an improvement in its compositional characteristics and nutraceutical profile due to FOC application [20,21]. The present study demonstrated that using FOC in the experimental GFB formula enhanced the nutritional value of the obtained bakery products. Compared with the control, all FOC-fortified GFBs were significantly (*p* < 0.05) enriched in proteins, which were proportional to the FOC level in the formulations. In particular, the protein content for FOC30% was eight times higher than in the control (Table 3). Flaxseed proteins are characterised by a beneficial amino acid composition, including arginine, aspartic acid, and glutamic acid [45], as well as cysteine and methionine, which have been linked to improved antioxidant status and potential health benefits [7]. Due to FOC, the experimental GFBs were also significantly (*p* < 0.05) enriched in fat and dietary fibre; however, this beneficial effect was detected only in bread with the highest FOC levels (FOC15% and FOC30%) (Table 3). High levels of fat in GFBs can potentially enhance the sensory attributes and increase satiety, but on the other hand, it may also result in reduced loaf volume and texture [46]. The use of FOC allowed for the enrichment of experimental GFBs with dietary fibre. Many commercially available GFBs are deficient in this important compound, which has beneficial physiological functions [25,47]; meanwhile, its adequate intake is important for maintaining gut health, regulating blood sugar levels, and reducing the risk of chronic diseases such as heart disease and diabetes [48]. The carbohydrate content of the GFBs decreased while the percentage of FOC increased (Table 3). Compared with the control, changes in the macronutrient content in FOC-fortified GFBs, particularly in terms of proteins and fat content, resulted in a reduction in the energy value.

### 3.2. The Content of Acrylamide in GFBs

The processing at temperatures >120 °C may intensify the progress of the Maillard reaction. The negative impact of acrylamide formation and its increased content in the final product as a result of heat treatment may be observed [49]. On the other hand, FOC contains asparagine (a domain precursor of acrylamide; 12.5 g/100 g of protein) [50], and therefore, the monitoring of acrylamide content in products with FOC as an additive is favourable. The acrylamide content in the analysed samples was below the LOD and LOQ. The maximum benchmark of acrylamide formation depends on the type of bread, e.g., for wheat bread it is 50 µg/kg, whereas for maize, oat, barley, spelt, and rice-based products, the limit is 150 µg/kg [51]. Because a very low amount of acrylamide was determined in the GFBs with FOC, it can be concluded that the addition of FOC at levels of 5, 15, and 30% is safe.

### 3.3. Physical Parameters and Crumb Colour

**Figure 1:**
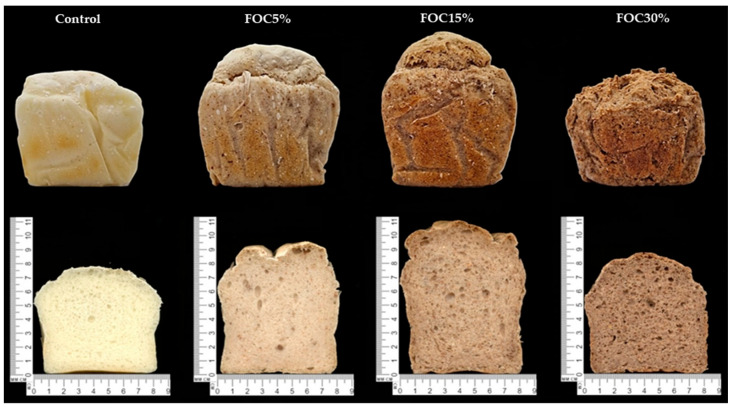
The surface and cross-section of experimental gluten-free breads.

The physical parameters, crumb colour, and appearance of the experimental GFBs are presented in Table 4 and Figure 1, respectively. The control GFB was dense and showed a low specific volume and height/width ratio (Table 4), which is a typical characteristic of a starchy GFB when compared with conventional wheat bread [52].

In general, the FOC used in the present study resulted in the improvement of a majority of the physical parameters of the experimental breads (Table 4; Figure 1). In comparison with the control, FOC5% and FOC15% were characterised by a significantly higher specific volume and height/width ratio, and their crumbs were of the lowest density. Contrarily, FOC30% was of a low quality, as indicated by the physical parameters that were analysed. The instrumental colour analysis showed that the crumb of all experimental GFBs with FOC was significantly (*p* < 0.05) darker compared with the light-creamy control (L* = 71.78) (Table 4; Figure 1). These results are consistent with the FOC colour analysis (L* = 49; a* = 6.3; b* = 20; data not published), indicating that the colour and amount of FOC influence the bread crumb colour. Moreover, the decrease in the L* value and W index was proportional to the increasing level of FOC in the experimental GFB formula, which confirmed the crumb darkening. The coordinates for the values a* and b* were positive in all FOC-enhanced GFBs and increased with the increasing addition of FOC, which resulted in a more yellow (a*) and red (b*) hue of the crumb. The obtained results indicated that the incorporation of FOC in moderate amounts had a positive impact on the physical parameters and colour of GFBs, bringing them closer to the characteristics typically found in conventional wheat bread [53]. Among the experimental breads, FOC15% showed a favourable crumb colour and the best technological quality.

### 3.4. Evaluation of Texture Profile

The texture of the crumb of starchy GFBs is denser and more compact than that of conventional wheat bread due to the absence of gluten, which provides elasticity and structure [52]. The texture parameters of GFBs are highly influenced by the ingredients used. The control was characterised by a hard crumb of low cohesiveness and resilience (Table 5). In the present study, FOC was incorporated as a potential texturizing component. The applied FOC affected the hardness of the experimental GFBs; however, the observed effect was dependent on the amount of this by-product in the formulation.

A low-to-moderate amount of FOC (5–15%) significantly (*p* < 0.05) reduced the crumb hardness, gumminess, and chewiness (Table 5). On the contrary, at 30% of FOC, the crumb texture quality decreased significantly. Compared with the control, the crumb of FOC15% was two-fold harder, and additionally, it was the most gummy and chewy of all the experimental breads. These findings suggest that FOC can be used as a texturizing agent, which is in agreement with earlier studies using flaxseed [21,54]; however, its amount in GFB formulations should be controlled to achieve the desired texture characteristics. The obtained results are consistent with previous studies that reported that FOC as a high-fat ingredient can negatively affect the texture profile [55,56]. Further studies may consider the use of supplementary texturizing agents or processing techniques to enhance the texture profile of the developed experimental GFBs, since bread texture is a critical factor influencing sensory quality and consumer acceptance.

### 3.5. Sensory Analysis

**Figure 2:**
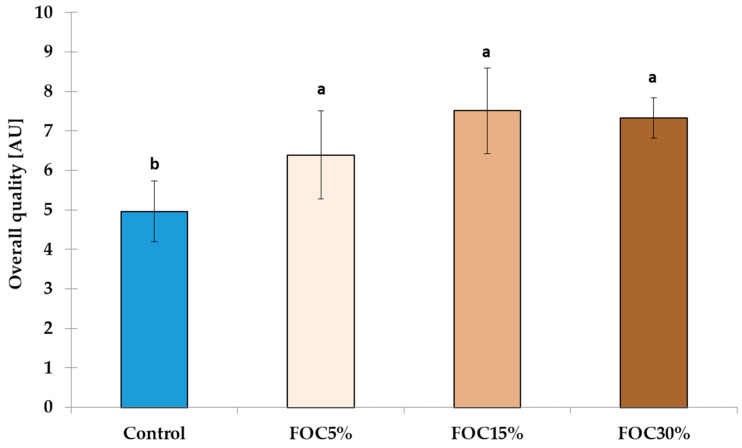
The overall sensory quality of experimental gluten-free breads. AU—Arbitrary Units; Different letters (a, b) above the bars represent significant differences (*p* < 0.05) between values, as determined via the one-way analysis of variance (ANOVA) and Fisher test.

When developing a new product, it is essential to conduct an analysis of the sensory quality. Therefore, the GFBs were assessed using a QDA (Table 6; Figure 2).

The aroma of the control bread was the most oily, sweet, and wheat bread-like but the least acid (Table 6). The application of FOC changed the aroma attributes of the breads. With an increasing amount of FOC, the GFBs became less oily, sweet, and wheat bread-like, but their acid aroma was more intense. In addition, they were characterised by a seed-like aroma that was not detected in the control (Table 6).

Regarding appearance, a creamy colour was only present in the control GFB, while a brown colour was found in all GFBs with FOC (Table 6). The intensity of this parameter was proportional to the amount of FOC in the formulation. The colour of the experimental GFBs mainly depends on the colour of the raw material used, and therefore, the colour differences between the control and GFBs with FOC results from the FOC shade, which was dark brown (L* = 49.13, a* = 6.25, b* = 20.18). Colour is the single most important product-intrinsic sensory cue that influences people’s expectations regarding the likely taste and flavour of food [57]. The darkening of the experimental bread due to FOC use is considered a desirable feature, because in general, GFBs are characterised by an unpalatable and light hue compared with their conventional equivalents [52]. The pore collocation values decreased with the rise in FOC content, indicating that the pores became more irregular. In contrast, the pore dimension values increased with the FOC percentage, indicating a larger pore size.

The application of FOC in the experimental formulation had a significant (*p* < 0.05) influence on the texture of the GFBs, both when analysed manually and in the mouth. All GFBs containing FOC were significantly (*p* < 0.05) more elastic, chewy, adhesive, and moist than the control (Table 6). Importantly, better texture parameters were obtained in the case of bread with a higher FOC content. These findings are consistent with the TPA results (Table 5), distinguishing FOC15% as a sample of the desirable texture, resulting from its softness and the lowest gumminess and chewiness.

In terms of taste, all experimental breads with FOC, regardless of their percentage, were characterised by a sweetness that was similar to the control and a slightly salty and oily taste (Table 6). In turn, a seed-like taste and bitter taste were detected in all FOC-enhanced GFBs, and their perceptibility increased with the increase in FOC content in the formulation.

All experimental GFBs with FOC had a significantly (*p* < 0.05) higher overall quality than the control (Figure 2); in particular, FOC15% was distinguished by the panellists as the best. The QDA demonstrated that the addition of FOC to GFBs had a beneficial effect on sensory attributes, which highlights the potential of using this by-product to improve the sensory quality of GFBs. In addition, the high overall quality scores of FOC15% are consistent with the previously described results of physical parameters (Table 4) and texture profiles (Table 5), which confirm its high technological quality. Nevertheless, further studies may be useful to determine the shelf life of the products. Moreover, it would be of great interest to conduct a consumer study assessing the sensory acceptability, which could provide valuable insights into the overall acceptability of the FOC-enriched GFBs, helping to determine their viability on the market.

## 4. Conclusions

The conducted study showed that FOC, due to its valuable characteristics, augmented the nutritional value of developed GFBs. The physico-technological parameters, colour, and texture of GFBs were beneficially modified by the incorporation of FOC. At low-to-moderate levels (5% and 15%), FOC improved the specific volume, texture characteristics (reduced crumb hardness, gumminess, and chewiness), and appearance of GFBs, which allowed us to ameliorate its sensory features, although a seed-like aroma and taste were noticed. However, as an increase in crumb hardness was detected with an increased FOC percentage, the concentration of FOC needs careful regulation to achieve the desired textural characteristics. Among the obtained experimental formulations, FOCE15% can be perceived as the most appreciated product due to its improved quality, providing an opportunity to meet the nutritional needs and sensory expectations of individuals following a gluten-free diet.

## Figures and Tables

**Table 1:** Composition of experimental gluten-free breads.

Ingredient (%)	Control	FOC5%	FOC15%	FOC30%
Corn starch	36.7	34.8	31.2	25.7
Potato starch	8.9	8.5	7.6	6.2
Pectin	2.2	2.2	2.2	2.2
Sugar	2.8	2.8	2.8	2.8
Salt	0.8	0.8	0.8	0.8
Rapeseed oil	1.4	1.4	1.4	1.4
Fresh yeast	2.8	2.8	2.8	2.8
Flaxseed oil cake	-	2.3	6.8	13.7
Deionised water	44.4	44.4	44.4	44.4

**Table 2:** Sensory attributes, their definition, and scale edges used in the descriptive analysis (QDA) of gluten-free breads with FOC.

Attribute	Definition	Scale Edges
Aroma		
Oily	Typical sunflower oil aroma	None–Very intensive
Sweet	Typical aroma of sweet baked products from wheat flour	None–Very intensive
Wheat bread	Typical aroma of wheat-baked products	None–Very intensive
Acid	Typical aroma of organic acids	None–Very intensive
Seed-like	Typical flaxseed aroma	None–Very intensive
Appearance		
Creamy colour	Colour intensity according to colour pattern RAL 085 90 10—scale value 3	Light–Dark
Brown colour	Colour intensity according to colour pattern RAL 060 30 20—scale value 10	Light–Dark
Pore collocation	Visual impression of bread crumb pore arrangement	Irregular–Regular
Pore dimension	Visual impression of bread crumb pore size	Small–Big
Texture (manual)		
Elasticity	The extent to which a piece of product returns to its original shape when pushed by a finger	Small–Big
Texture (in the mouth)		
Chewiness	Multiplicity of chewing the product to prepare it to swallow	Low–High
Adhesiveness	Degree of adhesiveness perceived when chewing the sample 10 times	Low–High
Moisture	Degree of amount of water in the product perceived when chewing the sample 10 times	Low–High
Taste		
Seed-like	Typical flaxseed taste	None–Very intensive
Sweet	Basic taste illustrated by sucrose dissolved in water	None–Very intensive
Salty	Basic taste illustrated by sodium chloride dissolved in water	None–Very intensive
Oily	Typical sunflower oil taste	None–Very intensive
Bitter	Basic taste illustrated by caffeine solution dissolved in water	None–Very intensive
Aftertaste	Lingering sensation after swallowing the sample	None–Very intensive
Overall quality		
Overall quality contains the sum of all attributes and their harmonisation		Low–High

**Table 3:** Nutritional composition and energy value of FOC and experimental gluten-free breads.

	FOC	Control	FOC5%	FOC15%	FOC30%
Moisture (%)	8.98 ± 0.14	46.37 ^a^ ± 0.13	46.98 ^a^ ± 0.41	47.19 ^ab^ ± 0.42	48.01 ^b^ ± 0.45
Protein (g/100 g DM)	30.46 ± 0.15	1.16 ^a^ ± 0.06	2.50 ^b^ ± 0.01	5.21 ^c^ ± 0.01	9.55 ^d^ ± 0.01
Ash (g/100 g DM)	4.79 ± 0.07	1.18 ^a^ ± 0.03	1.50 ^b^ ± 0.08	1.88 ^c^ ± 0.14	2.45 ^d^ ± 0.01
Fat (g/100 g DM)	15.89 ± 0.11	1.71 ^a^ ± 0.02	1.51 ^a^ ± 0.09	3.08 ^b^ ± 0.08	4.75 ^c^ ± 0.29
Dietary fibre (g/100 g DM)	7.88 ± 0.15	0.49 ^a^ ± 0.11	0.61 ^a^ ± 0.11	1.38 ^b^ ± 0.17	1.86 ^c^ ± 0.03
Carbohydrates (g/100 g DM)	32.00 ± 0.04	49.09 ^d^ ± 0.21	46.90 ^c^ ± 0.04	41.27 ^b^ ± 0.10	33.38 ^a^ ± 0.31
Energy value (kJ)		918 ^c^ ± 3	896 ^a^ ± 6	904 ^b^ ± 2	906 ^b^ ± 5
Energy value (kCal)		220 ^c^ ± 1	214 ^a^ ± 2	216 ^b^ ± 1	216 ^b^ ± 2

DM—Dry Matter; Values with the same letter (a, b, c, d) in each row do not differ significantly (*p* ≤ 0.05).

**Table 4:** Physical parameters and crumb colour of experimental gluten-free breads.

	Control	FOC5%	FOC15%	FOC30%
Specific volume (mL/g)	2.09 ^a^ ± 0.14	2.80 ^b^ ± 0.02	3.01 ^b^ ± 0.10	2.11 ^a^ ± 0.06
Bake loss (%)	10.99 ^a^ ± 0.10	13.05 ^b^ ± 0.36	13.72 ^b^ ± 0.41	11.03 ^a^ ± 0.34
Density (g/mL)	0.48 ^b^ ± 0.03	0.36 ^a^ ± 0.00	0.33 ^a^ ± 0.01	0.47 ^b^ ± 0.01
H/W ratio	0.95 ^a^ ± 0.09	1.10 ^b^ ± 0.05	1.27 ^c^ ± 0.03	0.95 ^a^ ± 0.10
Crumb colour				
*L*	71.78 ^d^ ± 0.92	68.07 ^c^ ± 0.49	57.27 ^b^ ± 0.59	47.98 ^a^ ± 0.20
*a*	−1.61 ^a^ ± 0.03	3.19 ^b^ ± 0.11	5.29 ^c^ ± 0.09	6.32 ^d^ ± 0.09
*b*	9.09 ^a^ ± 0.18	11.91 ^b^ ± 0.37	14.42 ^c^ ± 0.22	14.39 ^c^ ± 0.20
W index	70.31 ^d^ ± 0.91	65.77 ^c^ ± 0.38	54.59 ^b^ ± 0.50	45.65 ^a^ ± 0.21
ΔE	Served as control	6.69	16.93	25.65

Values with the same letter (a, b, c, d) in each row do not differ significantly (*p* ≤ 0.05).

**Table 5:** Texture profile of experimental gluten-free breads.

	Control	FOC5%	FOC15%	FOC30%
Hardness (N)	17.09 ^c^ ± 0.98	13.43 ^b^ ± 0.73	8.79 ^a^ ± 0.75	33.40 ^d^ ± 2.32
Springiness	0.98 ^a^ ± 0.01	0.98 ^a^ ± 0.02	0.95 ^a^ ± 0.04	0.97 ^a^ ± 0.03
Cohesiveness	0.34 ^a^ ± 0.01	0.39 ^b^ ± 0.02	0.40 ^bc^ ± 0.01	0.42 ^c^ ± 0.02
Gumminess	5.87 ^b^ ± 0.56	5.17 ^b^ ± 0.19	3.50 ^a^ ± 0.26	14.12 ^c^ ± 1.55
Chewiness	5.73 ^b^ ± 0.52	5.05 ^b^ ± 0.15	3.34 ^a^ ± 0.28	13.65 ^c^ ± 1.82
Resilience	0.14 ^a^ ± 0.01	0.17 ^bc^ ± 0.01	0.16 ^bc^ ± 0.01	0.18 ^c^ ± 0.02

Values with the same letter (a, b, c, d) in each row do not differ significantly (*p* ≤ 0.05).

**Table 6:** Results of the assessment of the sensory quality using quantitative descriptive analysis (QDA) in experimental gluten-free breads.

	Control	FOC5%	FOC15%	FOC30%	*p*-Value
Aroma					
Oily	1.98 ^a^ ± 0.99	1.62 ^ab^ ± 1.09	1.18 ^ab^ ± 1.10	0.85 ^b^ ± 0.79	0.0441
Sweet	2.79 ^a^ ± 0.75	1.95 ^b^ ± 0.43	1.44 ^b^ ± 0.93	1.45 ^b^ ± 1.25	0.0014
Wheat bread	2.93 ^a^ ± 0.79	1.65 ^ab^ ± 0.87	0.93 ^b^ ± 0.92	0.87 ^b^ ± 0.90	<0.0001
Acid	0.13 ^b^ ± 0.20	0.71 ^ab^ ± 0.58	1.02 ^a^ ± 0.56	1.29 ^a^ ± 0.50	<0.0001
Seed-like	0.01 ^c^ ± 0.01	2.08 ^b^ ± 1.39	3.55 ^ab^ ± 1.13	4.87 ^a^ ± 1.64	<0.0001
Appearance					
Creamy colour	3.43 ^a^ ± 0.97	0.01 ^b^ ± 0.01	0.01 ^b^ ± 0.01	0.01 ^b^ ± 0.01	<0.0001
Brown colour	0.01 ^c^ ± 0.01	1.93 ^bc^ ± 1.88	3.93 ^b^ ± 1.53	6.32 ^a^ ± 0.99	<0.0001
Pore collocation	7.88 ^a^ ± 0.92	5.94 ^ab^ ± 2.23	4.37 ^b^ ± 1.22	4.41 ^b^ ± 1.36	<0.0001
Pore dimension	1.13 ^b^ ± 0.48	1.76 ^b^ ± 0.85	3.71 ^a^ ± 1.23	3.35 ^a^ ± 0.68	<0.0001
Texture (manual)					
Elasticity	0.58 ^c^ ± 0.53	2.10 ^b^ ± 0.96	3.67 ^a^ ± 1.13	3.78 ^a^ ± 1.12	<0.0001
Texture (in the mouth)					
Chewiness	1.05 ^b^ ± 0.54	2.16 ^ab^ ± 0.73	3.04 ^a^ ± 0.85	3.33 ^a^ ± 0.99	<0.0001
Adhesiveness	0.78 ^c^ ± 0.23	1.71 ^b^ ± 0.44	2.64 ^a^ ± 0.42	2.94 ^a^ ± 0.74	<0.0001
Moisture	0.98 ^b^ ± 0.48	1.82 ^b^ ± 0.61	2.78 ^a^ ± 0.47	3.09 ^a^ ± 0.81	<0.0001
Taste					
Seed-like	0.01 ^d^ ± 0.01	1.91 ^c^ ± 0.65	3.68 ^b^ ± 0.74	5.07 ^a^ ± 1.02	<0.0001
Sweet	2.98 ^a^ ± 1.55	2.53 ^a^ ± 1.15	2.50 ^a^ ± 1.20	2.55 ^a^ ± 1.18	0.7725
Salty	0.29 ^a^ ± 0.22	0.34 ^a^ ± 0.26	0.38 ^a^ ± 0.24	0.33 ^a^ ± 0.23	0.8249
Oily	1.56 ^a^ ± 0.59	0.92 ^a^ ± 0.54	0.88 ^a^ ± 0.88	0.95 ^a^ ± 1.16	0.1577
Bitter	0.01 ^c^ ± 0.01	0.44 ^bc^ ± 0.56	0.63 ^ab^ ± 0.61	0.93 ^a^ ± 0.83	0.0031
Aftertaste	2.35 ^a^ ± 1.36	2.65 ^a^ ± 1.33	2.98 ^a^ ± 1.21	3.53 ^a^ ± 1.02	0.1303

Within each row and for each factor, values with the same letter (a, b, c, d) do not differ significantly (*p* ≤ 0.05) as determined through the one-way analysis of variance (ANOVA) and Fisher test. Differences between means were determined using the least significant difference (LSD) test.

## Data Availability

Data are contained within the article.
